# Recent Advances in PRRS Virus Receptors and the Targeting of Receptor–Ligand for Control

**DOI:** 10.3390/vaccines9040354

**Published:** 2021-04-07

**Authors:** Chia-Ming Su, Raymond Robert Richard Rowland, Dongwan Yoo

**Affiliations:** Department of Pathobiology, University of Illinois at Urbana-Champaign, Urbana, IL 61802, USA; cmsu2@illinois.edu (C.-M.S.); rowland7@illinois.edu (R.R.R.R.)

**Keywords:** porcine reproductive and respiratory syndrome, receptor, PRRSV, CD163

## Abstract

Cellular receptors play a critical role in viral infection. At least seven cellular molecules have been identified as putative viral entry mediators for porcine reproductive and respiratory syndrome virus (PRRSV). Accumulating data indicate that among these candidates, CD163, a cysteine-rich scavenger receptor on macrophages, is the major receptor for PRRSV. This review discusses the recent advances and understanding of the entry of PRRSV into cells, viral pathogenesis in CD163 gene-edited swine, and CD163 as a potential target of receptor–ligand for the control of PRRS.

## 1. Introduction

Infections caused by porcine reproductive and respiratory syndrome virus (PRRSV) emerged in the late 1980s in the United States and Europe almost simultaneously but independently [1]. PRRSV has quickly spread to most pork-producing countries worldwide and is responsible for one of the most economically important diseases to ever affect the global swine industry [2]. Recent taxonomy places the virus in the genus Betaarterivirus, subfamily Variarterivirinae, family Arteriviridae of the order Nidovirales (https://talk.ictvonline.org/taxonomy/p/taxonomy_releases, accessed on 25 February 2021). The family Arteriviridae now contains 23 species, including PRRSV, simian hemorrhagic fever virus (SHFV), lactate-dehydrogenase elevating virus (LDV), equine arteritis virus (EAV), and the newly recognized wobbly possum disease virus (WPDV). Two genotypes have been described for PRRSV: PRRSV-1 (European genotype; Betaarterivirus suid 1) and PRRSV-2 (North American genotype; Betaarterivirus suid 2). Both types cause a similar clinical disease but share only approximately 60% nucleotide sequence identity at the genome level [1,3,4,5,6]. Since its emergence, PRRSV has continually evolved. Some strains of PRRSV circulating in US swine herds in the late 1990s were found to be more virulent than those encountered in the past [7], and in 2007, highly pathogenic PRRSV-2 (HP-PRRSV) emerged in China, resulting in high mortality and severe respiratory clinical signs [8]. HP-PRRSV belongs to PRRSV-2, with only a few cases of PRRSV-1 [9,10]. In Europe, a highly pathogenic variant of PRRSV-1 was identified in Eastern Europe and named Lena virus [11]. The Lena virus is characterized by higher body temperature and more severe clinical signs compared with the Lelystad virus (LV) and other common field strains [12,13].

PRRSV virion is pleiomorphic. It is round or egg-shaped with a diameter of 50 to 74 nm according to the cryo-electron microscopy [14]. PRRSV is an enveloped virus containing a non-segmented, single-strand, positive-sense RNA genome. The genome is about 15 kb in length with a 5′-cap and 3′-polyadenylated [poly(A)] tail. The genome contains at least 10 open reading frames (ORFs) [15,16,17,18]. ORF1a codes for polyprotein 1a (pp1a). ORF1b is translated as a fusion protein with pp1a to yield polyprotein 1ab (pp1ab). The pp1ab polyprotein is produced as a result of a ribosomal frameshift by the presence of a pseudoknot and the slippery sequence. PP1a and pp1ab are further processed into 14 nonstructural proteins (nsp), including four proteinases: nsp1α (papain-like cysteine proteinase 1α or PLP1α), nsp1β (PLP1β), nsp2 (poliovirus 3C-like cysteine proteinase or CP), and serine proteinase (SP) in nsp4 [19]. Additional frameshifting events yield truncated nsp2TF and nsp2N products [20]. ORFs 2 through 7 encode four membrane-associated glycoproteins (GP2a, GP3, GP4, and GP5), three membrane proteins (Envelope (E), ORF5a, and Membrane (M)), and the nucleocapsid (N) protein [21].

The *Arteriviridae* have restricted host cell tropisms. LDV infects only mice and in culture; its replication is restricted to primary mouse peritoneal macrophages [22,23]. SHFV infects monkeys and in vitro; it is limited to simian primary macrophages and African green monkey kidney-derived cell lines, such as MA-104 [22]. EAV has a relatively broader tropism in cell culture, showing infectivity in BHK-21 (baby hamster kidney), HmLu (hamster lung), RK-13 (rabbit kidney), Vero (African green monkey kidney), LLC-MK2 (rhesus monkey kidney), MA-104, and MARC-145 (a derivative of MA-104) cells [24,25,26,27]. For PRRSV, *Suidae sus* is the only natural host, and in vitro, viral infection is limited to differentiated blood monocytes (BMo) and a subset of primary porcine alveolar macrophages (PAMs) [28,29]. MA-104 and MARC-145 are also susceptible to PRRSV and are commonly used for virus research in vitro and virus propagation for vaccines [30,31].

## 2. Viral Entry Mediators and Putative Receptors for PRRSV

At least seven cellular molecules have been proposed as entry mediators and putative receptors for PRRSV: CD169 (sialoadhesin; siglec-1), non-muscle myosin heavy chain 9 (NMHC II-A or MYH9), heparan sulfate, vimentin, DC-SIGN (CD209), CD151, and CD163 (cysteine-rich scavenger receptor) [19,32,33,34,35]. All of these receptors were initially characterized using in vitro model systems. The incorporation of studies using genetically modified pigs shows that CD163 is the only putative receptor that is necessary and sufficient for infection [36,37,38,39,40,41,42]. The precise role of CD169 remains unclear but may function as a co-factor for viral internalization [43,44]. MYH9 has recently been described as a receptor but requires further evidence independent of other investigators. Therefore, this review will primarily focus on CD163 as the main receptor for PRRSV.

### 2.1. PRRSV Entry Mediators

#### 2.1.1. CD169

Sialoadhesin, also referred to as CD169 or siglec-1, is a macrophage-restricted lectin that binds sialic acid. CD169 is a type I transmembrane glycoprotein belonging to the immunoglobulin superfamily possessing 17 extracellular Ig-like domain repeats followed by a short cytoplasmic tail [45]. CD169 expression is restricted to tissue macrophages, especially in secondary lymphoid tissues [46]. Expression in macrophages has been shown to facilitate host–pathogen interactions by promoting the uptake of sialylated pathogens, such as PRRSV [47,48], HIV-1 [49,50], Neisseria meningitidis [51], Campylobacter jejuni [52], and Trypanosoma cruzi [53]. Duan et al. [33] identified a 210 kDa protein involved in PRRSV infection of PAMs, which was later determined to be CD169 by internal peptide sequencing [48]. CD169 facilities the interaction between the macrophage and sialic acid on the PRRSV virion surface. Infection of cells can be blocked by sialoadhesin-specific mAbs, indicating that CD169 is essential for PRRSV infection of PAMs [54]. PRRSV non-permissive cells, such as PK-15, do not express CD169. However, when PK-15 cells were engineered to express porcine CD169, they became permissive for both PRRSV-1 and PRRS-2 internalization and uptake, suggesting the critical role of CD169 for viral endocytosis [48]. However, growth kinetics in the engineered cells showed that virus uncoating and replication were absent, suggesting that CD169 may function as a co-receptor [48]. Colocalization of CD169 and PRRSV virion on the cell surface and beneath the plasma membrane after infection further supports CD169 as an internalization mediator [44]. Analysis of gene expression patterns of CD169 in pigs demonstrates that transcription levels increase in the endometrial and placental macrophages after PRRSV infection [55], suggesting that CD169 plays a certain role in PRRSV infection in pigs.

CD169 as the receptor for PRRSV has been challenged by in vivo studies incorporating CD169 knockout (KO) pigs [56]. CD169 expression on PAMs was eliminated by removing part of exon 1 and all of exon 2 and exon 3 of the *SIGLEC1* gene. The absence of CD169 was confirmed by antibody staining of PAMs; however, CD163 remained intact. After inoculation with a PRRSV-2 isolate, viremia and antibody responses in the CD169-knockout pigs were similar to those in heterozygous or wild-type pigs, indicating that the absence of CD169 expression does not interfere with PRRSV infection [56]. The results describe the different outcomes that can be obtained when using in vitro versus in vivo model systems for investigating viral receptors. 

The exact role of CD169 in PRRSV infection remains unclear. CD169 may function as a co-receptor. Splenic CD163^+^ macrophages expressing a negligible level of CD169 are susceptible to PRRSV, indicating that a little of CD169 may be sufficient for PRRSV internalization [57]. Anti-CD169 antibodies block PRRSV infection of CD163^+^ macrophages, suggesting that CD169 still plays an important role in virus entry [57]. Recently, siglec-10, another sialic acid-binding immunoglobulin-type lectin, was found to improve PRRSV infection and production [58]. Interestingly, different strains of PRRSV show different infection preferences in PK-15 cells co-expressing siglec-1 and CD163, or siglec-10 and CD163 [59]. These results suggest that different genotypes and strains of PRRSV may preferentially utilize different siglec molecules.

#### 2.1.2. MYH9

Anti-idiotype antibodies are produced against the variable region of another antibody. Therefore, the anti-idiotype is a representation of the epitope recognized by the original antibody. The anti-idiotypic monoclonal antibody Mab2-5G2 was developed to recognize PRRSV GP5 protein [60]. This anti-idiotypic antibody was able to recognize a cellular protein in MA-104 cells and PAMs as the GP5 binding partner, and further study showed that this cellular protein was non-muscle myosin heavy chain 9 (MYH9) [34]. MYH9 is a motor protein involved in cell migration, adhesion, and morphogenesis [61]. The ectodomain of PRRSV GP5 interacts with the C-terminal domain of MYH9 during virus binding [34,62]. The ectodomain of GP5 induces aggregation of MYH9 and facilitates viral internalization in both MARC-145 cells and PAMs [62]. Further studies showed that the amino acids E1670, K1673, E1679, and I1683 in the MYH9 C-terminal domain are the key binding residues, and a point mutation in E1670 in PAMs causes reduced permissiveness for PRRSV infection [63]. Overexpression of S100A4, which is an MYH9 disassembly inducer, downregulates the MYH9 aggregation and results in the inhibition of both PRRSV-1 and PRRSV-2 infection [62]. Additionally, blebbistatin, which is the inhibitor of myosin II ATPase [64], blocks PRRSV infection in vitro and in vivo, which further confirms the role of MYH9 [34]. The soluble MYH9 C-terminal domain hinders the interaction with GP5 in a dose-dependent manner [65]. Further studies show that the MYH9 C-terminal domain interacts with the cysteine-rich scavenger receptor superfamily (SRCR) 1 through 4 regions of CD163 and facilitates PRRSV infection [66]. Recombinant CD163 SRCR1-4 inhibits infection of PRRSV-1 and PRRSV-2 in PAMs by competitive binding to MYH9 [66]. MYH9 may be a co-factor of CD163 for PRRSV infection.

#### 2.1.3. Other Mediators for PRRSV

*Heparan sulfate* is a highly acidic linear polysaccharide and belongs to the glycosaminoglycan family. Heparan sulfate is involved in various immune-associated activities, including leukocyte development, leukocyte migration, immune activation, and inflammatory processes [67]. It is expressed on the cell membrane and in the extracellular matrix of almost all mammalian cell types. Heparan sulfate has been demonstrated to serve as a receptor for several viruses, such as herpes simplex virus [68], human papillomavirus (HPV) [69], human immunodeficiency virus type 1 (HIV-1) [70], foot-and-mouth disease virus (FMDV) [71], and porcine circovirus 2 (PCV2) [72]. Heparan sulfate was identified as the potential mediator for PRRSV entry [73]. The proteoglycans of heparan sulfate and heparin-like molecules attach to the M protein and the M/GP5 complex of PRRSV [74]. Heparan appears to have a different role in the infectivity of different genotypes of PRRSV. However, heparan sulfate is not necessary for PRRSV infection of PAMs [74,75].

*Vimentin* is expressed on the MARC-145 cell surface and was identified to interact with the N protein of PRRSV [76]. Anti-vimentin antibodies block PRRSV infection, and the presence of vimentin converts non-permissive cells to cells that are susceptible to PRRSV infection. Vimentin may represent part of a larger PRRSV receptor complex.

*CD151* is a member of the transmembrane 4 superfamily. CD151 functions in cell signaling, cell activation, and platelet aggregation [77,78,79]. By screening a cDNA library for host proteins binding to 3’ UTR of PRRSV, CD151 was identified as an RNA-binding protein [80]. Overexpression of CD151 converted PRRSV non-permissive cells to permissive cells, indicating that CD151 may facilitate PRRSV infection. Treatment with an anti-CD151 antibody or gene silencing of CD151 expression inhibits the PRRSV infection [80]. Overexpression of a host microRNA (miR-506), which is known to downregulate CD151 mRNA and proteins, reduced PRRSV production in MARC-145 cells [81]. Although these studies propose that CD151 is a potent molecule regulating PRRSV infection, its role in PRRSV entry remains unclear.

*DC-SIGN (CD209)* is a human C-type lectin and has been found involved in the transmission of enveloped viruses [82]. BHK cells are PRRSV non-permissive, and over-expression of DC-SIGN enhances the transmission of PRRSV in trans, indicating that DC-SIGN may also take part in PRRSV infection [35].

### 2.2. CD163 as the Receptor for PRRSV

#### 2.2.1. CD163

**Figure 1:**
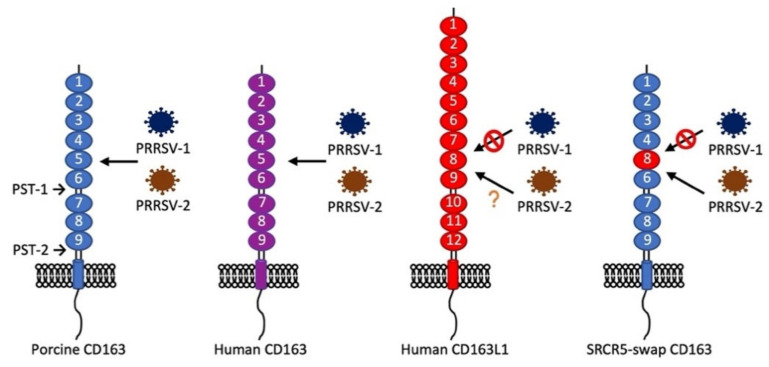
Structure of porcine CD163, human CD163, human CD163L1, and their supportability for porcine reproductive and respiratory syndrome virus (PRRSV)-1 or PRRSV-2 infection. Porcine CD163 supports PRRSV-1 and PRRSV-2 infections, whereas human CD163 supports PRRSV-2 infection [32]. Whether human CD163 supports PRRSV-1 infection is unknown. Human CD163L1 is a homolog of CD163 but does not support PRRSV-1 infection [94]. The cysteine-rich scavenger receptor superfamily (SRCR)5-swap CD163 was made by replacing the SRCR5 domain of porcine CD163 with the SRCR8 domain of human CD163L1. Expression of SRCR5-swap CD163 in HEK293T (human embryonic kidney cells) and porcine alveolar macrophages (PAMs) does not confer the permissiveness of PRRSV-1 [94,98]. However, SRCR5-swap CD163 in PAMs supports most PRRSV-2 strains infection [98], except PRRSV-2 JXA1 [99].

CD163 is a scavenger receptor expressed on the mature macrophages and monocytes and belongs to class B of the cysteine-rich scavenger receptor superfamily (SRCR-SF) [83]. CD163 consists of nine tandem repeats of the SRCR domain (SRCR1 through SRCR9), which are connected to the transmembrane domain and intracellular cytoplasmic tail. The functions of CD163 are the clearance of the cell-free form of hemoglobin (Hb) as well as participation in anti-inflammatory processes [84]. After hemolysis, the cell-free hemoglobin binds to haptoglobin (Hp) and forms the Hb–Hp complex. The SRCR3 domain of CD163 binds to the Hb–Hp complex, which subsequently removes the complex from the circulation by CD163-positive macrophages in the liver, spleen, and bone marrow [85]. The Hb–Hp complex is then transferred to the early endosomes in CD163-positive macrophages and is further degraded in lysosomes, while CD163 is recycled back to the plasma membrane [86]. The expression of CD163 is restricted to the monocyte/macrophage lineage and can be used as a differentiation marker for the maturation of tissue macrophages [87,88,89]. CD163 has been identified as the receptor for SHFV [90] and PRRSV [91]. The soluble form of CD163, which contains most of the extracellular domains, is detected in the circulation and body fluids and can be a biomarker for some clinical conditions, such as sepsis, autoimmune diseases, multiple sclerosis, and malaria [92]. By screening a cDNA expression library of PAM cells, CD163 was identified as important for PRRSV infection [32]. Subsequent experiments showed that the ectopic expression of CD163 made non-permissive cells permissive for PRRSV [32,93], demonstrating that CD163 is likely an essential molecule for PRRSV infection. To identify the specific domains of CD163 involved in PRRSV infection, the replacement and deletion of each domain of porcine CD163 were made, and each construct was further tested for infection with a PRRSV-1 isolate [94]. Human CD163L1 is a homolog of porcine CD163; however, human CD163L1 does not support PRRSV infection. Each domain of porcine CD163 was substituted with the corresponding domain of human CD163L1 to generate different chimeric CD163 constructs (Figure 1) [94]. After swapping the SRCR5 domain of CD163 with the corresponding domain (SRCR8) of human CD163L1, the chimeric construct was negative for PRRSV-1 infection, indicating that the SRCR5 domain of CD163 is essential for PRRSV invasion [94]. The CD163 proline-serine-threonine (PST)-rich region connects SRCR9 with the transmembrane domain, which is also required for PRRSV, but the exchange of PST with the homologous domain from human CD163L1 does not affect permissiveness [94], implying that the major function of the CD163 transmembrane domain is to anchor CD163 to the cell membrane [95]. CD163 has different isoforms and different sizes of intracellular cytoplasmic tails. The long-tail isoforms have no effect on CD163 biological functions [96]. Expressing a tailless form of CD163 enhances viral production by an unknown mechanism [97].

#### 2.2.2. In Vitro Evidence for CD163 as the Receptor for PRRSV

CD163 as the primary cellular receptor for PRRSV has been confirmed by numerous investigators. Non-permissive cell lines, such as BHK-21 (baby hamster kidney), PK-0809 (porcine kidney), NLFK (feline kidney), LLC-PK (porcine kidney), and PK-15 (porcine kidney), and 3D4/21 (immobilized porcine alveolar macrophages) are made permissive for PRRSV infection after overexpression of CD163 [32,93,100,101]. NPTr (newborn pig trachea) is a porcine epithelial cell line derived from newborn pig tracheal cells and is non-permissive to PRRSV but becomes permissive after overexpression of CD163 [102]. Murine alveolar macrophage-derived cells (MH-S) and murine peritoneal macrophage-like cells (RAW264.7) are widely used to study macrophage-specific immune properties in vitro. After expression of CD163, these cells became permissive for PRRSV [43]. Infected cells show pro-inflammatory cytokine expression profiles similar to infected PAMs [43]. Recently, porcine endometrium epithelial cells (PEC) were isolated and immortalized by expressing the SV40 large T antigen [103]. These cells express both CD163 and 169 and are susceptible to PRRSV infection, including the induction of apoptosis [103].

PAMs immortalized using SV40 large T antigen provide an opportunity to develop a continuous cell line for the propagation of PRRSV. A cell line designated 3D4/21 (ATCC CRL-2843) was successfully produced but is unexpectedly non-permissive for PRRSV growth [104]. Permissiveness was restored by the constitutive expression of porcine CD163 [101]. In addition, PRRSV replicated more efficiently in the pCD163-expressing 3D4/21 cells than in MARC-145 cells [101]. Recently, a novel, simple, and efficient PiggyBac (PB) transposon method was applied to 3D4/21 cells to express porcine CD163 [105]. Immortalized PAM cells were generated by stable expression of the human telomerase reverse transcriptase (hTERT) using a retrovirus vector [106]. The expression level of endogenous CD163 was not affected and the immortalized cells remained permissive for infection with PRRSV-1 and PRRSV-2 strains [106]. The functional relationship between CD163 and PRRSV infection has been confirmed by varying the abundance of CD163 in immortalized PAMs [107]. In this study, CD163-positive single-cell-derived clones were sorted based on levels CD163 expression. Only 20–34% of cells were CD163 positive. Further analysis showed that the infection rate was proportional to the abundance of CD163 on the cell surface. Cells expressing low levels of CD163 were non-permissive. Interestingly, CD169 expression was absent in the immortalized PAMs [107]. This finding further supports the hypothesis that CD163 is the primary receptor for PRRSV. Taken all together, the in vitro studies show that CD163 alone can convert non-permissive cells to cells permissive for PRRSV, including a productive replication cycle. 

Conversely, the removal of CD163 can make cells resistant to PRRSV. PAMs treated to suppress CD163 mRNA and surface expression using artificial microRNA (amiRNA)-expressing recombinant adenoviruses or amiRNA-containing exosome treatment confer resistance to different strains of PRRSV-2 [108]. Since silencing of CD163 mRNA in PAMs conferred resistance to PRRSV infection, the relation between CD163 and PRRSV was further studied in MARC-145 cells. Using a CRISPR/Cas9 gene editing strategy, the entire exon 7, which codes for SRCR5, was removed in MARC-145 cells. The modified cells showed complete resistance to PRRSV-2 infection [109]. Interestingly, localization of the virions in the early endosome was visualized at the beginning stage of infection in both wild-type and modified MARC-145 cells, but in the CD163 modified MARC-145 cells, localization of virions was only observed in the late endosome. Further examination of the interaction of CD163 and viral proteins shows that SRCR domain 5 deletion from CD163 inhibits the PRRSV uncoating in the early endosome by affecting the interaction of CD163 with GP2a, GP3, and GP5 [109]. 

Treatments that modulate CD163 surface expression also affect PRRSV permissiveness in cells. Surface expression of CD163 on PAMs and CD14-positive monocyte-derived macrophages (MDMs) can be modulated by treatment with interleukin (IL)-10, lipopolysaccharide (LPS), or tissue plasminogen activator (TPA) [93]. After treatment with TPA and LPS, CD163 expression on the cell membrane decreased, along with a reduction in PRRSV production [93]. Another study showed that treatment with LPS inhibited PRRSV infection in PAMs and MARC-145 cells [110]. Since the TLR4 (toll-like receptor 4)–NF-κB (nuclear factor-kappa B) pathway is activated in LPS-treated cells at the early stage of PRRSV infection, proinflammatory cytokines were strongly induced and subsequently reduced the CD163 expression. As a result, the CD163 downregulation led to the suppression of PRRSV infection. A disintegrin and metalloprotease 17 (ADAM17; also called TACE (tumor necrosis factor-α-converting enzyme)) also showed the ability to downregulate the expression of CD163 [111]. Overexpression of ADAM17 inhibited PRRSV infection by regulating CD163 expression, whereas the reduction of ADAM17 expression by siRNA led to upregulation of CD163, which further increased infection with PRRSV [112]. These findings demonstrate the positive correlation of CD163 expression and PRRSV infection.

#### 2.2.3. In Vivo Evidence for CD163 as the Receptor for PRRSV

**Figure 2:**
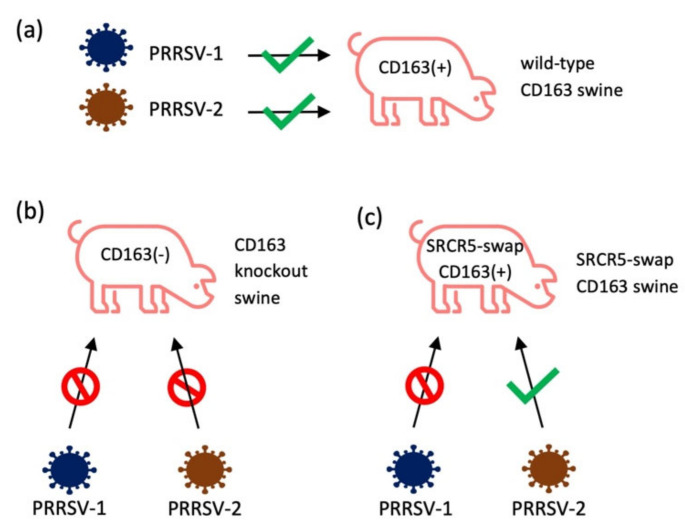
The gene-edited swine with CD163-KO or SRCR5-swap CD163 were tested for the permissiveness of different strains of PRRSV. (**a**) All strains of PRRSV-1 and PRRSV-2 can infect wild-type pigs; (**b**) after knockout CD163, swine were resistant to PRRSV-1 (SD16-15, Lelystad, 03-1059, 03-1060, SD01-08, 4353-PZ [98], H2, DAI, SU1-Bel [114], BOR-57 [38]) and PRRSV-2 (NVSL 97, KS-06, P129, VR2332, CO90, CO84, MLV-ResP, KS62, KS483 [98], TP [39], JXA1 [40], MY [41]); (**c**) SRCR5-swap CD163 swine were resistant to PRRSV-1 (SD16-15, Lelystad, 03-1059, 03-1060, SD01-08, 4353-PZ) [98]; however, these swine still minimally allowed PRRSV-2 infection (NVSL 97, KS-06, P129, VR2332, CO90, CO84, MLV-ResP, KS62, KS483) [98].

The role of CD163 for PRRSV infection has been studied in swine. By using the CRISPR/Cas9 gene-editing technology, pigs were genetically engineered [113], and the first CD163 gene-knockout (KO) pigs were generated (Figure 2b) [36]. After inoculation with the PRRSV-2 NVSL 97-7895 strain, the CD163-KO pigs showed no clinical signs, pathological changes, viremia, or antibody response. CD163 heterozygous fetuses were produced by mating boars with the CD163-KO gilts. While CD163 heterozygous fetuses remained PRRSV susceptible after birth, CD163-KO dams were able to protect fetuses from maternal infection with PRRSV [37]. Independent of this study, another group deleted the SRCR domain 5 (SRCR5) of CD163 by zygote injection of CRISPR/Cas9 and generated CD163 gene-edited pigs [38]. These pigs retained expression of the remaining portion of CD163 protein, and the biological activity related to removal of hemoglobin remained intact [38,114]. PAMs were isolated from these pigs and examined for their susceptibility to PRRSV. When placed in culture, PAMs from CD163-KO pigs were completely resistant to various strains of PRRSV, including six different isolates of PRRSV-1 and nine different isolates of PRRSV-2 [98]. PAMs and peripheral blood monocytes (PBMCs) were recovered from CD163 SRCR5-deletion pigs. The PAMs and PBMCs were fully resistant to PRRSV-1 and PRRSV-2 infection [114]. The SRCR5-deleted pigs were resistant to PRRSV-1 and showed no signs of infection, viremia, or PRRSV-specific antibody. There was no evidence of the presence of virus-infection in the lungs and lymph nodes compared with wild-type control pigs [38]. Two other research groups also developed CD163 SRCR5-deletion pigs. When infected with HP-PRRSV, these pigs were resistant to two different strains, TP [39] and JXA1 [40], indicating that the domain 5 of CD163 plays a critical role for PRRSV infection in pigs. 

Instead of deleting the entire SRCR5 of CD163, an attempt was made to delete a short region of SRCR5, which forms the ligand-binding pocket. These pigs were also completely resistant to PRRSV-2 JXA1 and MY strains [41]. These results support a role for the SRCR5 ligand-binding pocket region of CD163 in its interaction with PRRSV. Aminopeptidase N (APN) is the cellular receptor for transmissible gastroenteritis virus (TGEV). Double gene-knockout pigs were generated, which lacked CD163 and pAPN. Pigs were completely resistant to PRRSV-2 and TGEV infection, which demonstrates how pigs can be made resistant to more than one pathogen [42]. 

Since CD163 is the scavenger receptor for the hemoglobin–haptoglobin complex, the knockout of CD163 may result in negative physiological impacts on the host. To minimize this possibility, SRCR5 of porcine CD163 was replaced with SRCR8 of human CD163L1 (Figure 2) [98]. The pigs of complete knockout of CD163, deletions of SRCR domain 5, and SRCR5 domain swap pigs were examined for PRRSV susceptibility. First, PAMs from CD163-KO pigs were completely resistant to a panel of six PRRSV-1 and nine PRRSV-2 isolates. PAMs from SRCR5 domain-swap pigs were also resistant to PRRSV-1. However, these PAMs allowed the growth of PRRSV-2 [98]. This result is consistent with in vivo infection of SRCR5 domain-swap pigs [98]. Interestingly, an independent study in China showed that pigs with CD163 SRCR5 domain-swap with hCD163L1 SRCR8 were resistant to HP-PRRSV JXA1 infection [99]. Since HP-PRRSV JXA 1 is a PRRSV-2 strain [8], the variations among isolates within PRRSV-2 could produce different outcomes for SRCR5 domain swap experiments.

Besides the generation of gene-edited pigs, soluble receptors were used to understand the roles of CD163 and other putative receptors in PRRSV permissiveness. Soluble CD169 (Sn4D-Fc) and soluble CD163 (SRCR59-Fc) were expressed using adenovirus vectors (rAd) [115]. Pigs were inoculated with rAd–Sn4D-Fc and rAd–SRCR59-Fc followed by infection with PRRSV-2 JXA1. The rAd–Sn4D-Fc-treated pigs exhibited more severe clinical signs than the rAd–SRCR59-Fc-treated pig [116]. The co-expression of soluble CD169 and soluble CD163 provided complete protection against PRRSV infection [116]. These results confirm the hypothesis that CD163 is the core receptor for PRRSV.

Gene expression patterns for CD163 in the lung show that mRNA for CD163 is upregulated during PRRSV infection [117], providing further support for CD163 as the PRRSV receptor. The kinetics of PRRSV N protein and CD163 expression in the lungs and tonsils of PRRSV-1 infected piglets show that the number of CD163-positive cells initially decreased, but after 7 dpi increased until 35 dpi [118]. The initial decrease of CD163-positive cells is likely due to virus growth and cell death, and the recovery of CD163-positive cells may be due to the induction of CD163 expression in immature cells, the recruitment of CD163-positive cells to infection sites, or both.

## 3. Putative Viral Ligands for CD163

The PRRSV GP5 and M proteins were initially hypothesized as the ligands for receptors. To test this hypothesis, chimeric constructs were made to substitute GP5 and M from EAV with the respective proteins of PRRSV. The substitution of EAV GP5-M with PRRSV GP5-M did not affect the cell tropism of EAV [119,120]. Additionally, the substitution of the short ectodomain of PRRSV M with the EAV corresponding sequence did not change the cell tropism of PRRSV [121]. These results indicate that neither GP5 nor M are viral ligands and do not determine the cell tropism of PRRSV [119,120,121]. However, A Tyr10 deletion in the M protein conferred PRRSV resistance to a broadly neutralizing antibody [122]. Since the Tyr10 deletion is adjacent to a cysteine residue that mediates the disulfate bond formation with GP5 protein, this deletion may create a conformational change of M to regulate the virus–cell interaction [122].

GP2a and GP4 proteins were identified as interacting with CD163 [123]. Using a PRRSV infectious clone, a chimeric virus was created to substitute ORFs 2a through 4 with those of EAV and examined for its cell tropism [124]. The chimeric virus was unable to infect PAMs and possessed a cell tropism similar to EAV, indicating that GP2a, GP3, and GP4 play essential roles in PRRSV infection [124]. The glycans on GP2a and GP3 are required for PRRSV infectivity, and the glycosylation of GP2a and GP4 is an essential component for interaction with CD163 [125]. Amino acid substitutions in GP2a (V88F, M94I, F95L) are found in MARC-145 cell-adapted PRRSV strains [126]. Presumably, these substitutions may result in a stronger interaction with the SRCR5 domain of CD163.

## 4. Targeting the Receptor–Ligand for Control of PRRSV

Commercial vaccines including both modified live and inactivated vaccines are available for the control of PRRS. It is, however, generally accepted that the current vaccines are less satisfactory and that better vaccines are needed [127]. As a substitute, inhibiting the receptor–ligand interaction is a potential target for the control of PRRSV infection. Entry blockers have emerged as one of the antiviral strategies against PRRSV infection [128]. Blocking the interaction between virus and CD163 is an attractive target. One strategy is to develop a broad-spectrum antibody that can block the binding of PRRSV to CD163. The porcine CD163 SRCR5 protein structure has been determined by X-ray crystallography, and the 3-D structure shows the presence of a loop 5–6 region (Phe544-Arg570). An amino acid change in the loop 5–6 region of SRCR5 inhibits PRRSV infection [129]. Thus, antibodies or small molecules that can interact with a specific region of CD163 SRCR5 may block virus binding. Soluble SRCRs 5 through 9 of CD163 delivered by an adenoviral vector reduced PRRSV infection in PAMs [115], suggesting that a soluble receptor binds to PRRSV and prevents the receptor–ligand interaction and subsequently viral infection. Although SRCR5 appears to be the most important domain for ligand binding, the single SRCR5 domain is not sufficient to block the virus [115], and the remaining domains of CD163 may be necessary [94]. By replacing SRCR5 with SRCR8 of hCD163L1 in pigs, these animals remained completely permissive to PRRSV-2 but not to PRRSV-1 [98]. This study demonstrates that different genotypes of PRRSV may exhibit different mechanisms for the recognition of CD163. Since the CD163 SRCR5 domain is crucial for PRRSV infection, antibodies against this domain are of interest in blocking PRRSV entry. A monoclonal antibody against the SRCRs 5-6 region may partially block PRRSV infection in PAMs [130]. The target epitope for this mAb on SRCR5 is adjacent to the ligand-binding pocket. Other mAbs, 6E8 and 9A10, against SRCRs 5-9 showed a high activity for preventing PRRSV infection [131]. These mAbs showed dose-dependent inhibition of several strains of PRRSV-2 in PAMs and MARC-145 cells. Epitope mapping for 6E8 and 9A10 in CD163 binding show that they bind to the spanning residues of ^570^SXDVGXV^576^ in SRCR5 and Q^797^ in SRCR7, suggesting that multiple SRCR domains may be involved in PRRSV binding. Interestingly, 6E8 and 9A10 have different inhibitory efficiency against different PRRSV strains, suggesting that different viral proteins have different features in the dependence on CD163 [131]. By artificial intelligence molecular screening and cell-based bimolecular fluorescence complementation (BiFC) assay, a small molecule has been found to target SRCR5 of CD163 and has been shown to inhibit PRRSV infection of PAMs in a dose-dependent manner [132]. The linear polyethylenimine (PEI) also has the ability to inhibit PRRSV infection [133], although the mechanism for inhibition remains unknown.

## 5. Conclusions

PRRS is a complex disease, and some research data are confusing and conflicting. A better understanding of the precise mechanism for PRRSV entry will facilitate the design of new vaccines and antivirals. Recent advances in the receptor studies have revealed CD163 as the receptor. Ample evidence is available to demonstrate PRRSV takes advantage of using CD163 as the primary and core receptor and plays a role in the viral uncoating process. CD169 may be an accessory protein involved in viral internalization. There is a difference in how PRRSV-1 and PRRSV-2 strains recognize CD163, and there may be strain-dependent differences as well. With the phenotypic and genotypic diversities among PRRSV strains, virus binding and entry mechanisms may have evolved to be diverse. This may be a challenge to the development of a unitary strategy for antiviral drugs and vaccines. Further research will warrant the understanding of invasion mechanisms for PRRSV.

## Data Availability

Not applicable.
